# COVID-19 Deep Learning Prediction Model Using Publicly Available Radiologist-Adjudicated Chest X-Ray Images as Training Data: Preliminary Findings

**DOI:** 10.1155/2020/8828855

**Published:** 2020-08-18

**Authors:** Mohd Zulfaezal Che Azemin, Radhiana Hassan, Mohd Izzuddin Mohd Tamrin, Mohd Adli Md Ali

**Affiliations:** ^1^Kulliyyah of Allied Health Sciences, International Islamic University Malaysia, Bandar Indera Mahkota, 25200 Kuantan, Pahang, Malaysia; ^2^Kulliyyah of Medicine, International Islamic University Malaysia, Bandar Indera Mahkota, 25200 Kuantan, Pahang, Malaysia; ^3^Kulliyyah of ICT, International Islamic University Malaysia, 50728 Gombak, Kuala Lumpur, Malaysia; ^4^Kulliyyah of Science, International Islamic University Malaysia, Bandar Indera Mahkota, 25200 Kuantan, Pahang, Malaysia

## Abstract

The key component in deep learning research is the availability of training data sets. With a limited number of publicly available COVID-19 chest X-ray images, the generalization and robustness of deep learning models to detect COVID-19 cases developed based on these images are questionable. We aimed to use thousands of readily available chest radiograph images with clinical findings associated with COVID-19 as a training data set, mutually exclusive from the images with confirmed COVID-19 cases, which will be used as the testing data set. We used a deep learning model based on the ResNet-101 convolutional neural network architecture, which was pretrained to recognize objects from a million of images and then retrained to detect abnormality in chest X-ray images. The performance of the model in terms of area under the receiver operating curve, sensitivity, specificity, and accuracy was 0.82, 77.3%, 71.8%, and 71.9%, respectively. The strength of this study lies in the use of labels that have a strong clinical association with COVID-19 cases and the use of mutually exclusive publicly available data for training, validation, and testing.

## 1. Introduction

Opacity-related findings have been detected in COVID-19 radiographic images [1]. In one study [2], bilateral and unilateral ground-glass opacity was detected in their patients. Among paediatric patients [3], consolidation and ground-glass opacities were detected in 50%-60% of COVID-19 cases, respectively. This key characteristic may be useful in developing deep learning model to facilitate in screening of large volumes of radiograph images for COVID-19 suspect cases.

Deep learning has the potential to revolutionize the automation of chest radiography interpretation. More than 40,000 research articles have been published related to the use of deep learning in this topic including the establishment of referent data set [4], organ segmentation [5], artefact removal [6], multilabel classification [7], data augmentation [8], and grading of disease severity [9]. The key component in deep learning research is the availability of training and testing data set, whether or not it is accessible to allow reproducibility and comparability of the research.

One technique that is commonly used in deep learning is transfer learning which enables adoption of previously trained models to be reused in a specific application [7]. Established pretrained deep neural networks have been trained on not less than a million images to recognize thousands of objects as demonstrated in the ImageNet database [10]. The image set consists of typical and atypical objects, for example, pencil, animals, buildings, fabrics, and geological formation. One method of transfer learning is to freeze all layers except the last three layers—fully connected, softmax, and classification layers. The last three layers are then trained to recognize new categories. Pretrained models have shown promising results, in some instances, comparable with experienced radiologists [11].

Data quality is of paramount importance for a successful deep learning. “Garbage in, garbage out” colloquial applies as much to a general deep learning application as it does to deep learning in chest radiography. Previous research argues that radiologist interpretive errors originate from internal and external sources [12]. The examples of the former sources are search, recognition, decision, and cognitive errors, while the latter sources can be due to fatigue, workload, and distraction. Inaccurate labels used to train deep learning architecture will yield in underperforming models.

Recent research [11] has developed radiologist-adjudicated labels for ChestX-ray14 data set [4]. These labels are unique in the sense that they required adjudicated review by multiple radiologists from a group of certified radiologists with more than 3 years of general radiology experience. Four labels were introduced, namely, pneumothorax, nodule/mass, airspace opacity, and fracture.

With the recent opacity-related finding as an important characteristic in COVID-19 patients, this research is aimed at developing a deep learning model for the prediction of COVID-19 cases based on an existing pretrained model which was then retrained using adjudicated data set to recognize images with airspace opacity, an abnormality associated with COVID-19.

## 2. Methods

**Figure 1:**
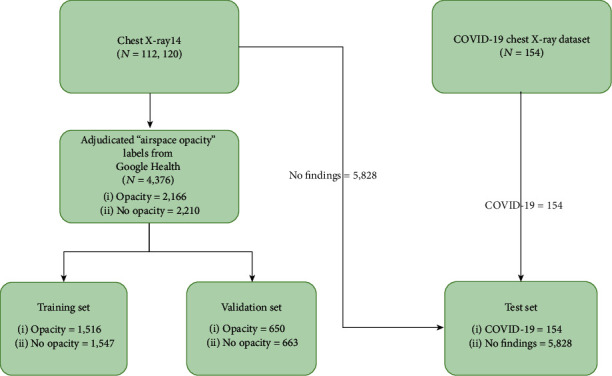
Flowchart of X-ray images used in this study. Training, validation, and test sets are mutually exclusive.

Independent sets were used for each training, validation, and testing phase. The training and validation data sets were extracted from ChestX-ray14 [4], a representative data set for thoracic disorders for a general population. The data set originated from the National Institutes of Health Clinical Centre, USA, and comprises approximately 60% of all frontal chest X-rays in the centre. The labels were provided by a recent research from Google Health [11]; the research was motivated by the need of more accurate ground truth for chest X-ray diagnosis. In this research, only one label was used to develop the deep learning model—airspace opacity, which is known to be associated with COVID-19 cases [1]. The COVID-19 cases in the testing data set were taken from COVID-19 X-ray data set, curated by a group of researchers from the University of Montreal [13]. Only frontal chest X-rays were used in this study. To simulate a population scenario with 2.57% prevalence rate, a total of 5828 images of “no finding” label from ChestX-ray14 were extracted to complement the test set.  Figure 1 summarizes the data sets used for the development and evaluation.

**Figure 2:**
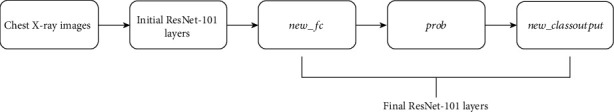
Initial and final layers of ResNet-101 deep learning network architecture employed in this study. All images need to be resampled to 224 px × 224 px × 3 channels to accommodate the network's input.

The depth of deep learning architecture is important for many visual detection applications. ResNet-101, a convolutional neural network with 101 layers, was adopted in this research due to its residual learning framework advantage that is known to have lower computational complexity than its counterpart, without sacrificing the depth and in turn the accuracy [14]. The network was pretrained on not less than a million images from a public data set (http://www.image-net.org/).  Figure 2 illustrates the initial and final layers of the network. Learning rates of all the parameters of all layers were set to zero except *new_fc*, *prob*, and *new_classoutput*, which refer to fully connected, softmax, and classification output layers, respectively. Only these three layers were retrained to classify chest X-ray images with airspace opacity. The network parameters were updated using stochastic gradient descent with momentum using options as tabulated in Table 1.

The *prob* layer outputs probability assigned to each label *j = {none, COVID-19}* which is defined as

(1) $$\begin{array}{c} p\left(y=j\;|\;x\right)=\frac{e^{\left(w_{j}^{\text{T}}x+b_{j}\right)}}{\sum_{j=1}^{k}e^{\left(w_{j}^{\text{T}}x+b_{j}\right)}}, \end{array}$$

where **x** is the output of the *new_fc* layer with transposed weights of **w**^T^ and bias *b*. The decimal probabilities of each instance must sum up to 1.0. For example, *image A* would have a probability of 0.8 that it belongs to label *none* and probability of 0.2 that it belongs to label *COVID-19*.

The *new_classoutput* layer measures the cross entropy loss for the binary classification with the following definition:

(2) $$\begin{array}{c} \text{cross entropy loss}=-\overset{N}{\underset{i=1}{\sum }}\overset{K}{\underset{j=1}{\sum }}t_{ij}\ln p\left(y\right), \end{array}$$

where *N* is the number of samples, *K* is the number of labels, and *p*(*y*) is the output from the *prob* layer.

## 3. Results and Discussion

**Figure 3:**
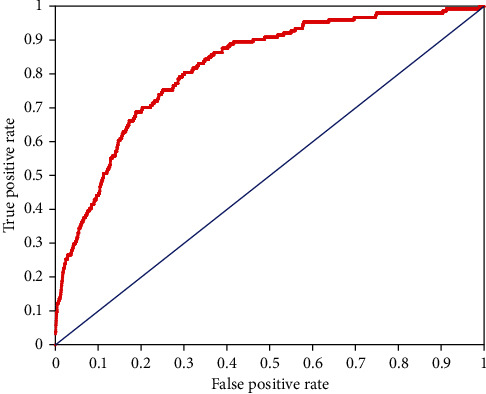
Receiver operating characteristic curve illustrating the performance of the deep learning model in predicting COVID-19 cases.

The performance of the model was evaluated using receiver operating characteristic curve as plotted in Figure 3. The area under the curve (AUC) was found to be 0.82, in which a value of 1.00 indicates a perfect COVID-19 test and 0.50 (as plotted by the blue line of no discrimination) represents a diagnostic test that is no better than random coincidence.

The published performance of deep learning models using radiographic images ranges from AUC = 0.82 to 0.996 [15–18]. Besides different deep learning methodologies adopted, modality and data set used also contribute to the variation in the performance. The study with the AUC = 0.996, for instance, used CT scan [15] as modality which generates higher resolution images compared to X-ray. Other studies using X-ray images use small number of images in their testing data set due to the fact that a significant portion of images were already used in the training phase [17, 18]. In addition, small data set can result in overfitting of the model to limited variation of COVID-19 cases.

**Figure 4:**
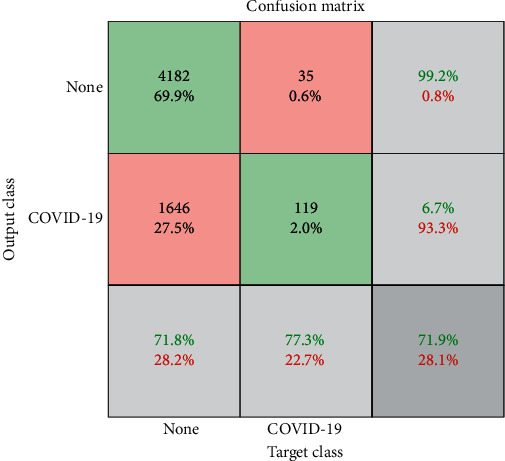
Confusion matrix of the deep learning model for COVID-19 classification using the testing data set.

**Figure 5:**
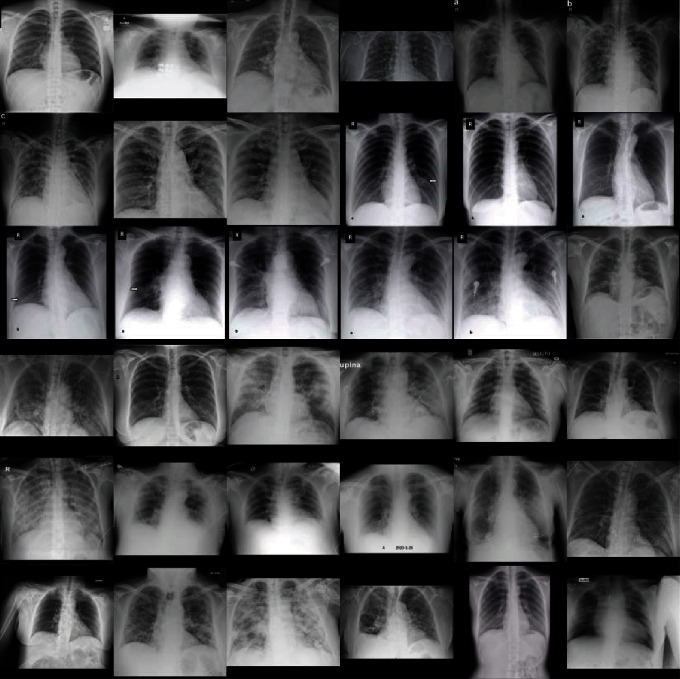
X-ray images with matched classification between deep learning model output and COVID-19 cases.

**Figure 6:**
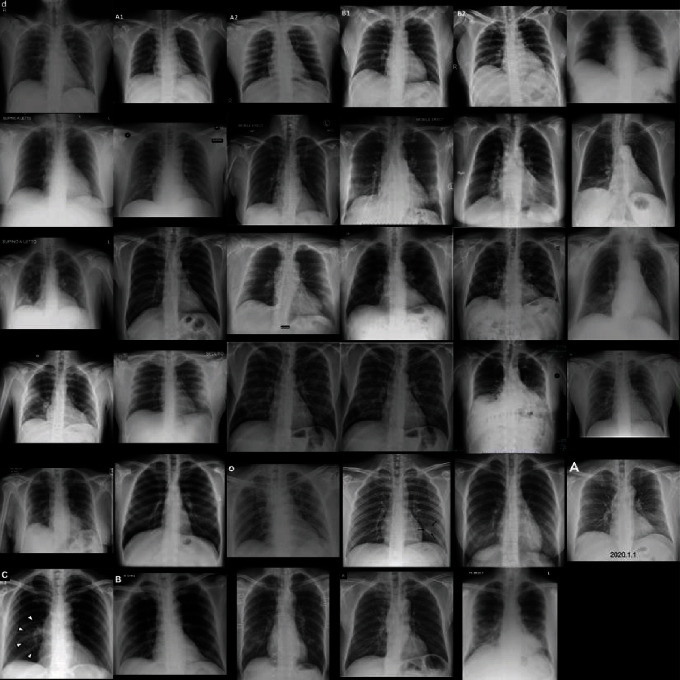
X-ray images with mismatched classification between deep learning model output and COVID-19 cases.

A confusion matrix was constructed in Figure 4 to summarize the binary classification performance of the model with the sensitivity, specificity, and accuracy of 77.3%, 71.8%, and 71.9%, respectively. Examples of true positive and false negative of COVID-19 cases are presented in Figures 5 and 6, respectively.

**Figure 7:**
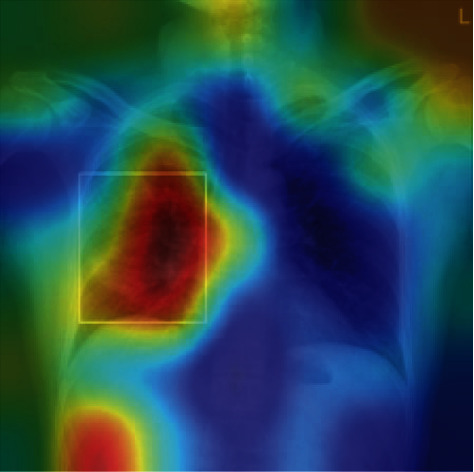
Class activation mapping algorithm can help to identify critical zones in the images; the deep learning model identifies what has been described by the radiologist as *“…patchy consolidation in the right mid lung zone.”*

Subjective validation of the model can be done by identifying the important zones in the image which contribute to the decision of the deep learning network. Gradient-weighted class activation mapping was used for this purpose [19]. The method determines the final classification score gradient with respect to the final convolutional attribute plot. The places where this gradient is high are precisely the places where the final score most depends on the results.  Figure 7 illustrates the important features highlighted by deep red colour and less relevant characteristics of the image depicted as deep blue.

Operational efficiency in radiology can be defined in terms of time taken to complete a task including imaging examination duration [20]. The research work, however, did not include the time required for the delivery of the final interpretive report. To estimate the operational efficiency of this model, we define a new parameter, adopting relative operational efficiency formula from the literature [21]:

(3) $$\begin{array}{c} \text{Model Efficiency}=\frac{\text{MPM}}{\text{RPM}}, \end{array}$$

where MPM is the number of images that can be processed by the model per minute and RPM is the number of images that can be processed by a radiologist per minute. Using the testing data set, MPM was estimated as 453 images per minute run on Intel® Core™ i7-4770 CPU. RPM, on the other hand, was estimated based on the radiologist average time to interpret the images with various pathologies, which was reported as 1.75 images per minute [22]. Based on these assumptions, the model was estimated to be 258 times more efficient than a radiologist. The model efficiency was significantly increased by four times when a GPU was used to accelerate computations.

A previous work [23] comparing ten convolutional neural network architecture using 1020 CT slices from 108 COVID-19 patients and 86 controls found that ResNet-101, which was also used in this current study, could achieve 99.02% accuracy. The work, however, employed a high-resolution CT scanner, which is not as ubiquitous as an X-ray imaging system. While the training and testing data were split, the images were sourced from the same data set which may lead to inaccuracy if the model is tested on images acquired from different CT scanners.

## 4. Conclusion

The strength of this study lies in the use of adjudicated labels which have strong clinical association with COVID-19 cases and the use of mutually exclusive publicly available data for training, validation, and testing. The results presented here are preliminary due to the lack of images used in the testing phase as compared to more than 1900 images in the testing set of an established radiography data set [11]. Deep learning models trained using actual COVID-19 cases can result in better performance; however, until and when adequate data are available to generalize the results of real-world data, cautionary measures need to be taken when interpreting the performance of the deep learning models applied in this context.

## Figures and Tables

**Table 1:** Options set for the network training.

Property	Options
Mini batch size	10
Maximum epochs	8
Initial learning rate	1*e* − 4
Shuffle	Every epoch
Validation frequency	Every epoch

## Data Availability

The data are available at https://github.com/ieee8023/covid-chestxray-dataset and https://cloud.google.com/healthcare/docs/resources/public-datasets/nih-chest.
